# Nutritional Bar with Potato-Based Resistant Starch Attenuated Post-Prandial Glucose and Insulin Response in Healthy Adults

**DOI:** 10.3390/foods9111679

**Published:** 2020-11-17

**Authors:** Vishnupriya Gourineni, Maria L. Stewart, Meredith L. Wilcox, Kevin C. Maki

**Affiliations:** 1 Global R&D, Ingredion Incorporated, 10 Finderne Ave, Bridgewater, NJ 08807, USA; maria.stewart@ingredion.com; 2 Midwest Biomedical Research, Addison, IL 60101, USA; mwilcox@mbclinicalresearch.com (M.L.W.); kmaki@mbclinicalresearch.com (K.C.M.); 3 Department of Applied Health Science, School of Public Health, Indiana University, Bloomington, IN 47405, USA

**Keywords:** resistant starch type-4, dietary fiber, capillary glucose, venous glucose and insulin

## Abstract

Resistant starch is a non-digestible starch fraction and is classified as fiber. Beyond naturally occurring fiber sources, starches can be modified to resist digestion, increase their fiber content and provide physiological benefits. The current study examined acute postprandial glycemic responses of VERSAFIBE™ 1490 resistant starch type-4, containing 90% total dietary fiber (TDF, AOAC (Association of Official Analytical Collaboration International) 991.43 method). In a double-blind, randomized, placebo-controlled, cross-over study, healthy adults (*n* = 38) consumed a nutritional bar containing either control (2 g), medium (21 g) or high (30 g) fiber. The test bars were matched with control for available carbohydrates, fat and protein. Venous glucose, insulin, and capillary glucose were measured. Mean ± SEM capillary glucose incremental area-under-curve (iAUC0)-120 min in min*mmol/L was lower (*p* < 0.005) for both fiber bars (136.2 ± 9.2 and 137.0 ± 10.4 for the medium and high fiber bars, respectively) compared to the control bar (174.9 ± 13.5). Mean venous insulin iAUC0-120 min in min*pmol/L was also lower for medium (8096.3 ± 894.5) and high fiber (7533.8 ± 932.9) bars, respectively, compared to the control bar (11871.6 ± 1123.9, *p* < 0.001). Peak capillary glucose and venous insulin concentrations were also significantly reduced (*p* < 0.001) after consumption of both fiber bars compared to the control bar. The results of this study suggest that nutritional bars containing potato based RS4 fiber reduced post-consumption glycemic and insulinemic responses when consumed by generally healthy adults.

## 1. Introduction

Health benefits of consuming a fiber-rich diet are well-established [1,2,3]. Dietary fibers are carbohydrates, which are not digested in the small intestine and are can be quantified using AOAC (Association of Official Analytical Collaboration International) method 2009.01 total dietary fiber method. Resistant starch is non-digestible carbohydrate or type of fiber and can also be measured using the Englyst method. To avoid confusion and communicate fiber levels on food labels, various national regulatory authorities have recommended to use integrated AOAC methods which measures all resistant starches and resistant polysaccharides [4]. However, physiological effects of starch can also be determined by its digestibility rate. Starch digestibility is determined using Englyst method, and are categorized into rapidly digestible (RDS), slowly digestible (SDS) and resistant starch (RS). Starch digestibility is dependent on intrinsic (structure, amylose content, amylose: amylopectin ratio, etc.) and extrinsic factors (processing, storage conditions etc.) [5,6]. Based on x-ray diffraction pattern which indicates amylopectin chain length and their packaging within a starch granule, cereal starches (waxy corn starch) have A-type crystalline pattern, while potato starch, raw banana starch have B-type crystallinity and C-type is seen in legumes and pulses [5]. During processing, digestible starches (RDS) can be modified to improve their nutritional/fiber profile and functionality resulting from the changes in the structural order of starch. This leads to reduced accessibility of starches to digestive enzymes (e.g., SDS and RS) [5].

The digestion rate of resistant starch has physiological implications such as in reducing post-prandial glycemic response in healthy [7], overweight or obese adults [8] and in populations at risk for type-2 diabetics [9]. Thus, replacement of digestible starch with resistant starch have beneficial glycemic effects in healthy and pre-diabetics [10,11]. In the European Union, resistant starch meets dietary fiber definition and European Food Safety Authority approved health claim which states—“replacing digestible starch with resistant starch induces a lower blood glucose rise after a meal” (at least 14% of the total starch content must be RS). This claim is applied to resistant starches from all sources [12].

Resistant starches are intrinsic in plants such as RS1 (physically entrapped starch) and are also isolated as an ingredient, including RS2 (granular with beta-crystals), RS3 (retrograded) and RS4 (chemically modified) [5]. A recent review [13] listed resistant starch content in foods commonly consumed in the US, including both processed and whole starchy foods. Most of the RS-containing foods are rich in fiber and thus can contribute to daily fiber intakes as per US dietary guidelines.

Resistant starch inclusion in commercial foods offer nutritional benefits (ex: fiber fortification, reduces caloric value), functional properties (e.g., improves texture) and organoleptic properties (e.g., crispiness). Thus, resistant starches are functional ingredients due to the presence of specific attributes such as pale color, bland flavor, fine particle size, high gelatinization temperature, low-water binding, good film forming and extrusion capabilities. These properties allow inclusion of resistant starches into variety of foods such as dairy, bakery and pasta. However, product development with resistant starches need careful consideration on rheological properties as types and sources of resistant starch have different impacts on finished products [1].

According to 2015–2020 Dietary Guidelines for Americans, dietary fiber is an under consumed nutrient of public health concern [14]. Based on the adequate intake (AI) of fiber dose (14 g/1000 Kcal) set by Institute of Medicine (IOM), the revised daily value for dietary fiber is 28 g/d for adults. Consumers can increase dietary fiber intakes by consuming whole grains, vegetables, fruits, nuts, legumes and pulses. Other fiber sources include commercial foods fortified with fiber or resistant starch. Over the years, commercial foods formulated with RS2 and RS3 were marketed [15]. More recently, RS4 ingredients are developed offering cost-effective solutions for fiber fortification. RS4 is chemically modified resistant starch, which is not available naturally in foods, but can be added as a functional ingredient [15]. In EU, native starches which are modified by physical, chemical or enzymatical treatments are considered as normal ingredients. Under EFSAs (European Food Safety Authority) novel foods provision, a chemically modified RS4, distarch phosphate starch are safe ingredients at maximum level of 15%, when added to low-moisture foods such as baked products, pasta, cereals and snacks [1]. In the US, RS2 and RS4 are approved as dietary fibers based on their physiological benefit on glycemic health [16].

RS4 ingredients are produced from various crops such as potato, tapioca, corn and wheat through different modifications such as dextrinization, etherification, esterification, acetylation, oxidation and cross-linking [17]. The resistance to digestion of RS4 is dependent on the type and extent of chemical modification [18]. For example, VERSAFIBE™ 1490 resistant starch is a distarch phosphate derived from potato that has been modified and its resistance to digestion is due to the presence of diester phosphate crosslinks within and between starch molecules [7].

There are few studies demonstrating glycemic health benefits of commercially available RS4. In a recent study, VERSAFIBE^TM^ 1490 potato based RS4 (distarch phosphate cross-links) when formulated in a cookie replaced digestible carbohydrates by delivering 24 g of fiber, reduced post-prandial glucose and insulin in healthy adults [7]. Similarly, another commercial RS4 made from tapioca starch, delivered 32 g of fiber in breakfast bar and resulted in beneficial reduction of post-prandial glycemic response compared to control bar [19]. Both of the aforementioned studies utilized substitution/replacement approach to reduce available carbohydrates and achieve post-prandial blood glucose management.

The new study-design guidelines in the US recommends macronutrient-matching of test foods with appropriate control food to demonstrate post-prandial glucose response as one of the physiological benefits of a fiber ingredient [20]. This approach was used by the researchers to validate nutritional bar formulated with wheat based RS4 (80 g RS4 or 20 g fiber) and matched with control bar for available carbohydrates, protein and fat. The results showed reduced post-prandial glucose and insulin in healthy adults [21]. However, in a dose-response study with VERSAFIBE^TM^ potato-based RS4 incorporated in a bar at 10 g and 20 g of fiber and matched for available carbohydrates did not impact post-prandial blood glucose and insulin in healthy adults [22]. Thus, it is important to understand the factors impacting these differences on post-prandial glycemic and insulemic response measurements. Thus, the present study aims to determine acute post-prandial glycemic and insulinemic response to a nutritional bar containing VERSAFIBE^TM^ 1490 resistant starch, an RS4, delivering a 21 g (medium) and a 30 g (high) fiber dose compared to a control bar (2 g fiber) in healthy adults. The primary outcome measured was capillary glucose incremental area-under-curve (iAUC0-120 min) for capillary glucose. The secondary outcome variables were the differences between fiber bars and control bar on venous glucose iAUC0-120 min and venous insulin iAUC0-120 min.

## 2. Materials and Methods

### 2.1. Study Design

The study was conducted according to Good Clinical Practice Guidelines, the Declaration of Helsinki (2000) and the United States 21 Code of Federal Regulations. An institutional review board (Hummingbird IRB, Needham, MA) approved the protocol before initiation of the study, and subjects provided written informed consent before any study procedures were performed. The study included one screening visit and three test visits.

### 2.2. Subjects

Inclusion criteria: healthy men and women aged 18 to 65 years, body mass index (BMI) 18.50–32.00 kg/m^2^, normally active and judged to be in good health on the basis of their medical histories, were enrolled in the study. As reviewed by Brouns et al. [23], subject characteristics, including BMI and glucose tolerance status do not appear to have a significant effect on relative glycemic responses, although groups differ in variability of response. Therefore, healthy human volunteers were included in the study covering a range of age and BMI values. Based on NHANES data from 2015–2016, a BMI of 32.0 kg/m^2^ represents the 71st percentile in the United States’ population.

Exclusion criteria: subjects were excluded if they had prediabetes (fasting capillary glucose between 100 and 125 mg/dL) or diabetes at screening, or any other clinically significant medical conditions. Subjects were excluded if they had fasting capillary glucose ≥100 mg/dL at screening; major trauma or a surgical event within 3 months of screening; history of drug or alcohol abuse; have body weight change ≥ 4.5 kg in the 2 months prior to screening; uncontrolled hypertension; use of antibiotics; symptoms of an active infection; intolerance to any ingredients in the study products; extreme dietary habits; cannot abstain from consuming probiotics; alcohol; smoking, and who are unwilling to comply with the experimental procedures.

### 2.3. Randomization and Allocation

The study was a randomized, double-blind, controlled, cross-over study with 41 healthy adults. At Visit 2, eligible participants were assigned to receive the three bars in random order on test days. A randomization schedule was generated using randomly permuted blocks and subjects were assigned to one of six sequence groups. There was a window of 2–28 days between the test visits.

At each test visit, a bar was consumed in its entirety with 8 oz. of water. Subjects were allowed to consume an additional 6 oz. of water following study product consumption at the first test visit (Visit 2), if desired. Ten minutes of total consumption time were allowed for ingestion of the study product and water. Subjects replicated their Visit 2 water/no water choice at Visits 3 and 4. Water was also allowed ad libitum after t = 60 min, where t = 0 min was the start of study product consumption. Subjects recorded all food and beverages consumed 24 h prior to Visit 2 and were instructed to replicate food and beverage selections to the best of their abilities during the 24-h period prior to Visits 3 and 4 for consistency.

### 2.4. Study Foods

**Figure 1:**
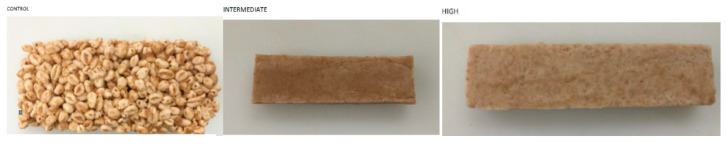
Cold pressed bars: control (**left**), medium-fiber (**middle**) and high-fiber (**right**).

The nutrient composition and formulation of the cold pressed bars are shown in Table 1 and Figure 1. The cold-pressed test bars contained 2 g (control), 21 g (medium) and 30 g (high) fiber from VERSAFIBE™ 1490 resistant starch, respectively (Ingredion Incorporated, Bridgewater, NJ, USA), which was the primary source of fiber. VERSAFIBE™ 1490 resistant starch is a resistant starch type 4 with 90% dietary fiber (AOAC 991.43). VERSAFIBE™ 1490 resistant starch is produced from food grade potato starch. The nutritional (fiber and control) bars were matched for fat, protein, and available carbohydrates. A small difference (around 1–2 g) between control and fiber bars is likely to have no impact on physiological response. Nutrient composition of the bars was calculated using Genesis R&D Food Labeling Software (ESHA Research, Salem, OR, USA). The fiber test bars were identical in appearance, but control bar was different in appearance due to usage of puffed wheat in the control bar (Table 2). This approach is similar to previously published bar formulation that tested wheat-based RS4 starch [21]. USFDA (The United States Food and Drug Administration) approved this formulation approach where control and test bars may appear different but must match available carbohydrates in both formulations. The formulation matching available carbohydrates of both test and control foods is acceptable by the US-FDA for evaluating post-prandial glucose response of an ingredient in order to qualify the ingredient as a dietary fiber per new fiber regulations [20]. The bars were packaged in an opaque enveloped with an alpha-numeric code for identification. Neither the study subjects nor the investigators knew the identity of the bars.

### 2.5. Biochemical Analysis

Capillary Glucose. The TRUE METRIX PRO Blood Glucose Meter (Trividia Health Inc., Fort Lauderdale, FL, USA) was used for determination of fasting capillary glucose at screening and on test days at fasting (t = −5 min [−15 to −2 min acceptable]) and t = 15, 30, 45, 60, 90 and 120 ± 2 min, where t = 0 min was the start of test bar consumption. Capillary glucose was measured immediately after the venous blood draws.

Venous Glucose and Insulin. At Visits 2–4, venous samples for the measurement of plasma glucose and insulin concentrations were collected at t = −5 min (−15 to −2 min acceptable) and at t = 15, 30, 45, 60, 90, and 120 ± 2 min, where t = 0 min was the start of study product consumption. The Cleveland Heart Lab (Cleveland, OH, USA) conducted the plasma glucose and insulin analyses. Glucose was measured using an enzymatic assay and insulin was measured with an electrochemiluminescence immunoassay.

Both capillary and intravenous blood samples were taken because previous studies indicated differences in blood glucose outcomes depending on the sampling technique. Capillary glucose has been shown to be more sensitive and offer greater precision over venous blood glucose, thus was selected for the primary analysis of glycemic response [23,24].

### 2.6. Statistics

#### 2.6.1. Sample Size Calculation

An evaluable sample of 34 subjects was expected to provide 80% power, alpha = 0.05, to detect a difference between treatments of 0.55 standard deviations in the primary outcome variable (G*Power version 3.1, Heinrich Heine University, Dusseldorf, Germany, Cohen’s d method). A sample of 41 subjects was randomized to allow for attrition.

#### 2.6.2. Data Analysis

The primary outcome was the difference between conditions in the incremental area-under-curve from 0 to 120 min (iAUC0-120 min) for capillary glucose. The secondary outcome variables were the differences between conditions on venous glucose iAUC 0-120 min and venous insulin iAUC0-120 min. For the iAUC calculation of the capillary and venous glucose and venous insulin measures, the pre-consumption measurement at the t = −5 min time point was counted as t = 0. Incremental area under the curve (i.e., area above the pre-consumption value without subtraction of area for values below the pre-consumption value) was calculated using the trapezoidal rule [24].

All tests of significance were assessed at alpha = 0.05, 2-sided. Statistical analyses were conducted using IBM SPSS Statistics for Windows, Version 25.0 (IBM Corp., Armonk, NY, USA). Differences in responses between study products were assessed using repeated measures analysis of covariance models with terms for treatment, sequence, and period as fixed effects and with subject as a random effect.

## 3. Results

A sample of 41 subjects was randomized. Subject characteristics are shown in Table 3 for all evaluable subjects, i.e., those who completed at least the control and one other condition. 

**Figure 2:**
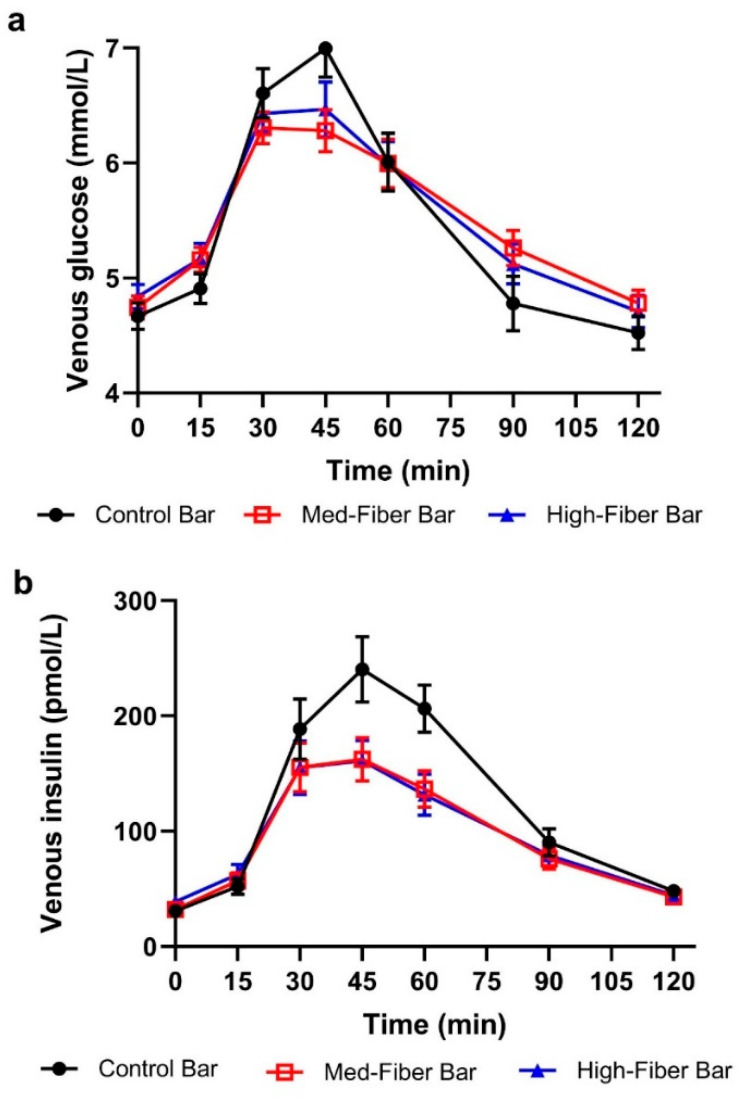
Post-prandial glycemic response in healthy adults (**a**) venous glucose (**b**) venous insulin concentration. Data are mean ± SEM. *n* = 37 (control); *n* = 36 (med fiber); *n* = 37 (high fiber).

**Figure 3:**
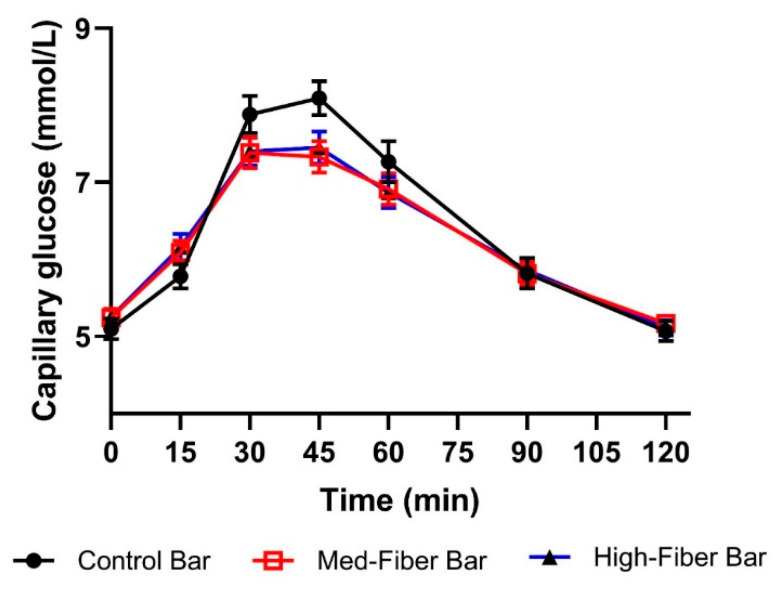
Post-prandial glycemic response (capillary glucose) in healthy adults. Data are mean ± SEM. *n* = 38.

Post-prandial mean venous glucose and insulin in response to control and test fiber bars are shown in Figure 2a,b. As shown in Table 4, there was no significant reduction in mean venous glucose iAUC in response to fiber bars compared to the control bar. However, venous insulin iAUC was significantly reduced with fiber bars post-prandially (*p* < 0.001) as compared to the control. Fiber bars significantly reduced post-prandial capillary glucose iAUC compared to the control, as depicted in Figure 3 and Table 4.

Capillary glucose iAUC0-120 min was lower for both the 21 g and 30 g fiber bars compared to the 2 g (control) fiber bar (*p* < 0.005 for both comparisons). Venous glucose iAUC0-120 min did not differ significantly among the three products (*p* = 0.263). Venous insulin iAUC0-120 min values lower for the 21 g and 30 g fiber bars, respectively, compared to the 2 g fiber (control) bar (*p* < 0.001 for both comparisons). After consumption of fiber bars, significantly lower peak values for venous glucose and venous insulin were observed as compared to control bar.

## 4. Discussion

According to the Centers for Disease Control and Prevention (CDC), approximately 34 million Americans have diabetes, adding burden to the health care costs and the US economy [25]. Due to rising health concerns, there has been a shift in consumer attitudes towards dietary choices, including functional foods [26,27]. Although, consumer inclination for nutritional foods has heightened, the reality shows intakes short of recommended fiber intakes globally. To fill this fiber gap, novel fiber ingredients and food product innovations have expanded the availability of fiber-fortified foods in the market. In the bakery segment, resistant starch has gained prominence as evident from the research published on different resistant starch applications in baked goods such as muffins, cookies, bread and cake [15].

The health benefits of resistant starch are well-established in maintaining digestive and glycemic health [4,28]. Post-prandial metabolism depends on how starch is digested into glucose and absorbed physiologically. Consumption of resistant starch can not only reduce post-prandial glucose and insulin but also impacts positively on other functions (inflammation, gut hormonal activity, etc.) through bacterial fermentation in the intestine [1,29,30].

Few studies have explored the glycemic effects of RS4 in baked goods applications. The current study investigated acute post-prandial glucose and insulin response of VERSAFIBE™ 1490 resistant starch (RS4) in healthy adults. Healthy participants consumed nutritional bar formulated with two fiber doses (21 g and 30 g), which were matched with control bar for available carbohydrates, protein and fat. The results show that the fiber bars significantly reduced capillary glucose iAUC 0-120 min and peak capillary glucose values, as well as venous glucose Cmax compared to the control bar. However, the venous glucose iAUC in response to fiber bars was not different from the control bar. This result is not surprising since capillary glucose measurements have been shown to be more sensitive for detection of differences in relative glycemic responses [23,24,31].

The results from the present study are consistent with those from another study [7] that utilized same potato fiber/RS4 at 24 g in a cookie. In that study, the fiber test cookies were formulated by replacing digestible starch fraction with resistant starch, so reduced glycemia was likely due, in part, to consumption of less available carbohydrate, whereas available carbohydrate was matched in the present study. In another recently published study [22], potato fiber/RS4 was assessed for dose-response effects on acute-post prandial glucose and insulin levels in healthy adults. This study tested potato fiber at 10 g and 20 g in bars that were matched for available carbohydrates, protein, and fat with a control bar. In contrast to the results from the present study, the authors reported no impact of potato fiber on glycemia at either dose compared to the control with. Potential factors that may have played a role in the variation in responses across studies include formulation (replacing digestible starch vs. matching available carbohydrates), baking conditions, food matrix, dosage of RS4, type and amount of available carbohydrates and portion sizes of the nutritional bars.

The source, structure and processing of resistant starch may have different effects on functionality in formulations, nutritional properties, and physiological functions. In a randomized, controlled, cross-over study, acute post-prandial glucose, and insulin response to wheat-based RS4 (20 g fiber or 80 g of RS4) was tested by matching macronutrients with the control bar. The results indicated that RS4 attenuated glycemic and insulinemic response even when high glycemic ingredients were consumed [21]. While in another randomized, controlled, cross-over study, tapioca-RS4 (32 g fiber) replaced digestible carbohydrates in a breakfast bar and significantly reduced post-prandial capillary glucose and venous insulin in healthy adults [19]. These results are consistent with those from a study of acid-hydrolyzed and heat-treated maize/corn RS4 formulated in baked muffin top (11.6 g fiber) and scones (16.5 g fiber), in which postprandial glucose and insulin responses were lower than low-fiber controls with similar total carbohydrate contents [31,32]. These differences of RS4 in ready-to-eat baked formulations (bar, muffin-tops, scones and cookie) may be attributed to RS4 sources, type of cross-linking, dose levels and product development approaches (i.e., matching available carbohydrates).

Mechanisms that could possibly explain the blood glucose and insulin lowering effects of VERSAFIBE™ 1490 resistant starch may be attributed to its structure and processing. The presence of cross-linked starch chains inhibits granular swelling, preserves granular integrity (preventing enzyme access), and creates steric hindrance, making amylase unable to properly bind to starch [15]. Possible mechanisms of resistant starch on glycemic management have been explained [1]. If test products are unmatched to control products for available carbohydrates, then decreased glycemic response is likely due to less available carbohydrate content. Alternatively, if available carbohydrates are matched in test and control products, a reduction in glycemic response may be due to reduced digestion or absorption of available carbohydrates [1]. However, these mechanisms need to be confirmed to show consistent effects in populations with normal and impaired glucose tolerance.

In the present study, potato based RS4 elicited lower post-prandial glucose and insulin in healthy adults, when formulated by replacing digestible starch with 21 g or 30 g fiber and matching available carbohydrates in a nutritional bar. The thermal stability of RS4 offers cost-effective solutions for fiber fortification in various products. The potato-based RS4 tested in this study is a certified low-FODMAP (low-Fermentable, Oligo, di, mono and polyols), offering greater intestinal tolerance to fiber in populations with intestinal sensitivity. The low-FODMAP diet is emerging as a first line of therapy for those suffering from irritable bowel syndrome (IBS), a disorder which is becoming a global phenomenon [33,34]. FODMAP’s are fermentable oligo, di, mono and polyols, commonly found in fruits, vegetables, legumes and nuts. Consumption of FODMAPs leads to gastrointestinal symptoms such as abdominal pain, bloating and altered bowel movements. IBS causes are multifold—could be resulting from bacterial fermentation of FODMAPs, their osmotic effects due to poor absorption and visceral sensitivity of gut. Dietary fiber continues to be important nutrient in daily diet. However, populations with intestinal hypersensitivity and IBS patients cannot consume fibers which are high in FODMAPs. Thus, fibers which have low-FODMAP’s such as resistant starches (RS) can be formulated to offer low-FODMAP food and beverages to IBS patients and populations with gut sensitivity [35]. Resistant starch from various sources (potato, tapioca and corn) qualify as low-FODMAP ingredients. The potato-based RS4 has higher tolerance as indicated by no change in composite gastrointestinal score in healthy adults [36].

The current study confirms acute post-prandial effects of potato based RS4 in healthy population. The study limitations include duration (short-term), population tested (healthy) and lack of confirmatory mechanisms to explain the observed results when available carbohydrates were identical in both control and test foods. Thus, future research may consider evaluating glycemic effects of RS4 for longer duration, with a focus to understand underlying mechanisms in both healthy and diabetic populations. 

## 5. Conclusions

In summary, capillary glucose and venous insulin iAUCs from 0 to 120 min were significantly lower after consumption of the 21 g and 30 g fiber bars compared to the 2 g fiber (control) bar. There were no differences among the three bars on venous glucose iAUC from 0 to 120 min, although the results were directionally similar to those for capillary glucose. The study demonstrates beneficial glycemic effects of potato based RS4 in a healthy study sample. RS4 incorporation in foods such as baked products, offer cost-smart approach to product developers seeking ways to increase fiber and improve functional attributes in ready-to-eat products. Consumers too will benefit from foods that will help meet their daily fiber requirements and aid in glycemic health management.

## Figures and Tables

**Table 1:** Nutrient composition of control and test bars.

Nutrient Content (g)	Control Bar	Med-Fiber Bar	High-Fiber Bar
Serving size	60	80	90.4
Total carbohydrate	37.61	56.29	64.55
Available carbohydrate *	35.60	35.23	34.86
Dietary fiber (RS)	2.01	21.06	29.69
Protein	6.16	4.13	4.07
Fat	2.18	2.23	2.12

* Available carbohydrates = total Carbohydrates—dietary fiber.

**Table 2:** Control and test (fiber) bars formulation.

Ingredient (g)	Control Bar	Med-Fiber Bar	High-Fiber Bar
Puffed wheat ^1^	25.1	0.0	0.0
VERSAFIBE™ 1490 RS4 ^2^	0.0	33.9	43.0
Wheat germ ^3^	25.9	18.0	15.3
Corn syrup ^4^	22.4	20.0	16.5
Brown sugar ^5^	8.2	11.0	8.3
Water	10.9	10.0	10.3
Gum acacia ^6^	6.5	6.0	5.0
Emulsifier ^7^	1.1	1.0	0.8
Glycerine ^8^	0.0	0.3	0.9

^1^ Quaker Oats, Barrington, IL, USA; ^2^ Ingredion Incorporated, Bridgewater, NJ, USA; ^3^ Kretschmer, Tukwila, WA, USA; ^4^ ACH Food Companies, Memphis, TN, USA; ^5^ Domino Foods, New York, NY, USA; ^6^ TIC Gums, White Marsh, MD, USA; ^7^ Dansico, New Century, Kansas, USA; ^8^ Global health repackage, Newark, New jersey, USA.

**Table 3:** Subjects Characteristics.

Variable	Participants (*n* = 38) Mean ± SEM or *n*
Age (yrs.)	46.07 ± 2.30
Gender (M/F)	20/18
Weight (kg)	77.3 ± 2.09
Body mass index (kg/m^2^)	26.2± 0.55
Fasting capillary glucose (mmol/L)	5.29 ± 0.74
Fasting venous glucose (mmol/L)	4.78 ± 0.74

**Table 4:** Incremental area-under-curve (iAUC) and peak values for glucose and insulin.

Outcomes	Control	Med-Fiber	High-Fiber	*p*-Value
Capillary glucose	174.9 ± 13.5 ^a^	136.2 ± 9.2 ^b^	137.0 ± 10.4 ^b^	0.002
Capillary glu peak	8.6 ± 0.23 ^a^	7.9 ± 0.18 ^b^	7.9 ± 0.19 ^b^	<0.001
Venous glucose	114.9 ± 10.4	102.6± 8.6	98.2 ± 10.8	0.263
Venous glu peak	7.4 ± 0.2 ^a^	6.8 ± 0.2 ^b^	6.9 ± 0.2 ^b^	0.008
Venous insulin	11871.6 ± 1123.9 ^a^	8096.3 ± 894.5 ^b^	7533.8 ± 932.9 ^b^	<0.001
Venous insulin peak	292.1 ± 29.5 ^a^	204.6 ± 19.6 ^b^	206.1± 22.32 ^b^	<0.001

iAUC, incremental area under the curve 0–120 min; glucose iAUC (min*mmol/L), capillary and venous glucose peak (mmol/L); insulin iAUC (min*pmol/L), insulin peak (pmol/L); data are mean ± SEM; *p*-values derived from repeated measures analysis of variance (ANOVA). ^a b c^ indicate significant differences across the control and test.
